# What makes a ‘successful’ collaborative research project between public health practitioners and academics? A mixed-methods review of funding applications submitted to a local intervention evaluation scheme

**DOI:** 10.1186/s12961-020-00671-0

**Published:** 2021-01-20

**Authors:** Peter van der Graaf, Lindsay Blank, Eleanor Holding, Elizabeth Goyder

**Affiliations:** 1 School of Health and Life Sciences, Teesside University, Centuria Building, Middlesbrough, TS1 3BA United Kingdom; 2 University of Sheffield, Sheffield, United Kingdom

**Keywords:** Decision-making, Public health, Qualitative research, Research personnel, Translational medical research

## Abstract

**Background:**

The national Public Health Practice Evaluation Scheme (PHPES) is a response-mode funded evaluation programme operated by the National Institute for Health Research School for Public Health Research (NIHR SPHR). The scheme enables public health professionals to work in partnership with SPHR researchers to conduct rigorous evaluations of their interventions. Our evaluation reviewed the learning from the first five years of PHPES (2013–2017) and how this was used to implement a revised scheme within the School.

**Methods:**

We conducted a rapid review of applications and reports from 81 PHPES projects and sampled eight projects (including unfunded) to interview one researcher and one practitioner involved in each sampled project (*n* = 16) in order to identify factors that influence success of applications and effective delivery and dissemination of evaluations. Findings from the review and interviews were tested in an online survey with practitioners (applicants), researchers (principal investigators [PIs]) and PHPES panel members (*n* = 19) to explore the relative importance of these factors. Findings from the survey were synthesised and discussed for implications at a national workshop with wider stakeholders, including public members (*n* = 20).

**Results:**

*Strengths*: PHPES provides much needed resources for evaluation which often are not available locally, and produces useful evidence to understand where a programme is not delivering, which can be used to formatively develop interventions. *Weaknesses*: Objectives of PHPES were too narrowly focused on (cost-)effectiveness of interventions, while practitioners also valued implementation studies and process evaluations. *Opportunities*: PHPES provided opportunities for novel/promising but less developed ideas. More funded time to develop a protocol and ensure feasibility of the intervention prior to application could increase intervention delivery success rates. *Threats*: There can be tensions between researchers and practitioners, for example, on the need to show the 'success’ of the intervention, on the use of existing research evidence, and the importance of generalisability of findings and of generating peer-reviewed publications.

**Conclusions:**

The success of collaborative research projects between public health practitioners (PHP) and researchers can be improved by funders being mindful of tensions related to (1) the scope of collaborations, (2) local versus national impact, and (3) increasing inequalities in access to funding. Our study and comparisons with related funding schemes demonstrate how these tensions can be successfully resolved.

## Background

Collaborative research projects between public health practitioners (PHP) and researchers are encouraged to increase the use of evidence in practice and decision-making. However, little is known about how to make collaborative research projects successful.

Previous research consistently suggests that research evidence is more likely to be used if users are engaged with researchers in defining the purpose and design of new research [1–8]. In particular, ‘sustained interactivity’ between researchers, policymakers and practitioners to support ongoing exchange, opportunities for personal two-way communication and partnership approaches is seen as important for making these partnerships work [5, 9].

Interpersonal trust and ongoing communication channels have been identified as essential to the process of developing close collaboration between research producers and users [10]. Long-term commitments from, and sustainable funding for, research is required to build these relationships over time [2]. In contrast, short-term initiatives are unlikely to work given the likely pace of organisational change and scale of the challenges facing academia and local government.

We have previously identified the need for an increased mutual awareness of the structures and challenges under which PHPs and researchers work [11]. Opportunities for frequent and meaningful engagement between PHPs and researchers can help to overcome barriers to co-production of evidence. Collaborative models, such as the use of researchers embedded in practice, might facilitate this; however, flexible research funding schemes are needed to support these models.

The difficulties for collaborative research have been well documented in studies of knowledge transfer and knowledge exchange in health services [1–3, 5, 12]. These studies consistently demonstrates that there is no single consistent definition of what constitutes ‘evidence’. This ambiguity results in inconsistencies in terms of what is used and valued as research between PHPs and academics. Particularly in local authorities, the use of research and evidence is highly political, with prevailing ideologies shaping the way evidence is identified, interpreted and considered at a local level [13, 14]. Linked to alignment with political ideologies, the timing of research is a key challenge for academics [15]. Research must be timely to fit with the notion of being able to influence and impact upon a specific ‘policy window’ and for evidence to be available when policymakers are likely to be receptive [16].

Experiences from various Collaborations for Leadership in Applied Health Research and Care (England) to develop collaborative research in health demonstrate that these issues are persistent and require constant alignment of relationships, values, structures and processes for collaboration, with a need for developing a shared 'collaborative' identity and new communities within existing networks that provide bridges across organisational boundaries [17, 18].

These experiences also suggest an ongoing need for dedicated funding programmes and spaces for researchers and PHPs to work together to generate research findings of greater utility to public health practice. Several research organisations in England have started to implement new services and programmes to create such opportunities.

One example is the Public Health Practitioner Evaluation Scheme (PHPES) run by the National Institute for Health Research School for Public Health Research (NIHR SPHR). PHPES [19] is a national, competitive scheme that offers PHPs support to evaluate local interventions in collaboration with SPHR researchers. The scheme was introduced in 2013 by SPHR to give access to researchers in its member organisations, which comprise eight leading public health research centres in England. PHPES aims to produce high-quality evidence needed by PHPs to improve population health and reduce health inequalities. PHPs can apply to the scheme for SPHR members to evaluate their local public health interventions. The scheme particularly focuses on local, rather than national, public health initiatives that have not been the subject of previous robust evaluations but which have potential applicability elsewhere and have secured operational funding for the research period.

No research exists to date on the evaluation of this scheme, and any assessment of the 'success’ of the scheme as a whole, or of individual projects, is complicated by the different priorities of stakeholders. To researchers, a successful proposal may be one that is funded; a successful project for researchers may be one that generates peer-reviewed journal papers or an impact case study, while a successful evaluation for many stakeholders is one that ‘proves’ a programme or intervention works. There might be additional disagreement between stakeholders as to whether generating evidence that an intervention does not achieve the intended outcomes or that suggests an intervention should be discontinued might equally constitute a successful evaluation. Clarifying from the start of collaborative applications what the shared expectations about ‘success’ are could therefore be crucial in achieving success from their different perspectives.

This paper reports on the evaluation of the NIHR SPHR PHPES and considers what makes collaborative research applications successful, or not, in the eyes of different stakeholders. We identify three tensions between practitioners and researchers that need to be resolved to maximise the potential for generating an impact on public health through these partnerships.

## Methods

The study consisted of four work packages with the overall purpose of making recommendations to the SPHR executive on the scope and implementation of a future responsive research fund:

1. A rapid review of applications and reports from PHPES projects (2013–2017).
2. Detailed review of applications and reports of a sample of eight projects (including funded and unfunded projects), and semi-structured telephone interviews with at least one researcher and one practitioner involved in each sampled application/project.
3. Online survey of practitioners (applicants), researchers (principal investigators [PIs]) and PHPES panel members (academic, practitioner and lay reviewers).
4. National workshops with a wide range of PHPES stakeholders (including lay representatives/community members and Public Health England [PHE]).

The research was conducted over a 9-month period between April and December 2019. The four work packages are discussed in more detail below. The findings from each work package informed the design of the data collection tools in the next work package to maximise data integration and facilitate an iterative research design.

### Work package 1: rapid review of all 81 applications and the project reports from 14 funded projects (April–June 2018)

The purpose of the review was to understand the scope of applications and more specifically to identify their original objectives in relation to generating generalisable findings and their dissemination/implementation. The review aimed to identify any common factors that are associated with (i) a successful application, (ii) an effectively delivered project and (iii) evidence of early impact (see Additional file 1: File 1 for the data extraction template used in the document analysis).

### Work package 2: individual in-depth interviews (mainly via telephone/Skype) (July–August 2018)

We sampled eight varied projects (from all applications potentially including funded and unfunded projects). The sampling frame/selection criteria were developed based on the review of documentation including applications and project reports. We selected six successful and two unsuccessful applications with representation from each SPHR members, aiming for a spread across topics and SPHR programmes. We interviewed one researcher and one practitioner involved in each sampled application/project. The lead researcher for each application was contacted first and, when they agreed to be interviewed, was asked for the contact details of their key practice partner, who was then approached for interview. In total, 34 researchers and practitioners were approached for interview; 14 people did not respond to the invitation and reminders (7 practitioners and 7 researchers), three people (2 academics and 1 practitioner) were not available for interviews during the fieldwork period, and two people had left the organisation they were working for at the time of their PHPES project and could not be reached.

Topic guides for practitioners and researchers (see Additional file 1: Files 2 and 3) included factors that make for a successful application, potential tensions between responsiveness and generalisability, and mechanisms for impact on policy and practice. Interviews were audiotaped and transcribed and a thematic analysis undertaken using NVivo software. To ensure we captured the breadth of relevant views on the programme, we also shared these initial findings and sought views from academic colleagues at Imperial College London, who were not part of the SPHR at that time, and from the chair and members of the PHPES panel which reviewed applications.

Telephone interviews lasted between 30 and 75 min, with an average of about 45 min. All interviews were audio-recorded and transcribed verbatim. Transcripts were coded and analysed thematically using a coding framework [20] informed by the interview schedule and themes drawn from published literature. Verbatim quotes from participants are included to illustrate the main themes identified.

### Work package 3: online survey of all applicants and their Local  Authority (LA) colleagues not involved in applications, PHPES researchers, PHPES reviewers and SPHR executive members (September–October 2018)

The online survey was developed based on work package (WP) 1 and 2 findings; it aimed to elicit views on the factors that influence applications/effective delivery/dissemination of evaluations and impact on policy and practice. Additional questions collected information on future research needs and priorities. The survey was modelled on a template developed for another SPHR evaluation project which reviewed public involvement in the first five years of the School [21]. The project’s public advisers contributed to the development of the survey, which also drew on interviews with SPHR researchers and members of the public involved in research. The template was adapted for use in this study to reflect the themes emerging from the interviews (see Additional file 1: File 4).

The online survey was circulated widely among SPHR members, including the executive group, advisory board and administrators in each member organisation, with a request to cascade to their policy and practice partners and patient and public involvement (PPI) panels to ensure that a range of additional perspectives on the issues identified in the interviews was included and to identify any additional issues arising from projects not sampled. In spite of wide circulation of the email invitation and an email reminder two weeks before the closing date of the survey, only 19 completed questionnaires were returned. Given that the circulation included a large number of stakeholders (estimated at around 100–200), the survey response rate was 10–20% at most. Because of the low response rate and likely response bias, with responses more likely from those with the most experience with the programme, responses cannot be interpreted as reflecting overall views of stakeholders. The responses do however provide useful insight into the views of a wider range of stakeholders, in addition to the evidence from those directly involved in developing proposals and delivering projects.

### Work package 4: exploring the implications of the study findings (September 2018)

Informed by findings from the first two work packages, a national workshop was organised in Sheffield to which a range of PHPES stakeholders (including lay representatives/community members/public health practitioners and other LA colleagues/PHE) were invited to discuss the implications of the findings and how they might inform a future responsive funding scheme. Through a series of interactive discussions, closely facilitated by the research team, participants were invited to reflect on future selection criteria, such as contributions to major public health problems, potential for impact and scalability, and assessment of evaluability, and on suggestions for co-producing knowledge within PHPES projects and measuring their impact on policy and practice (see Additional file 1: File 5 for the programme of the national workshop).

## Results

The Public Health Practice Evaluation Scheme (PHPES) was conceived as a response mode-funded evaluation programme. Fourteen PHPES projects were supported during the first five years of the SPHR’s work, covering a wide range of topics, types of public health programmes, and evaluation designs and methods. During the first SPHR programme, between 2013 and 2017, there were 81 applications made to the PHPES, from which the 14 funded evaluations were selected by a national panel. All funded projects were delivered, although a number were delayed or needed to adapt their methods for practical reasons, including changes in the delivery of the evaluated interventions. Evidence of early impact on local practices and policy was identified in some project reports. The success rate of applications and the sampling of projects across work packages is summarised in Table 1 below. Although it is inevitably difficult to ascribe causality, and there is often a lack of specific evidence in Local Authority (LA) policy or commissioning documents, there was evidence of policy changes or roll-out of programmes both during and after evaluations. For example, the Sheffield Housing+ programme addressed operational issues identified by the evaluation and the roll-out of the Better Care Fund’s St Ives Falls Prevention Pilot in Cambridgeshire, which also happened during the pilot evaluation.

**Table 1:** Success rates of applications/projects sampled in each work package

	Submitted applications	Successful applications	Projects delivered	Evidence of early impact
WP1	81	14	14	14^a^
WP2 (*n* = 15; 10 academics; 5 practitioners)	8 (11 participants from funded projects)	6 (4 participants from unfunded projects)	Not applicable^b^	Not applicable^b^
WP3	19 respondents	8 with previous PHPES experience	Not applicable^b^	Not applicable^b^
WP4	*n* = 15	Not applicable	Not applicable^b^	Not applicable^b^
Total	81	14	14	14

^a^All delivered projects identified local impact in their project reports

^b^No data were available on this indicator for the sampled projects in this work package

### Document analysis

The initial analysis of 81 PHPES applications/projects for the document review illustrated a wide range of projects but also highlighted gaps in the collection of data about projects (such as lack of details on partners, funding and theme). Despite emphasis in the guidelines on the importance of prior contact with SPHR partners, none of the applications for funded projects provided details about support received from SPHR members, and the majority of applications did not record prior contact with SPHR researchers before submission.

Most proposals were evaluations of interventions that were newly established (24 out of 44 for which data were available). ‘Changing Behaviour’ was the most popular theme for applications (23 out of 47), with ‘Identifying cost-effective population health services’ being the least popular (7 out of 48). Themes were based on existing research programmes and cross-cutting themes with SPHR [21]. All SPHR members were listed as partners on applications, with Sheffield being the most prolific (8/43) and Cambridge and London School of Hygiene and Tropical Medicine being the least involved, with one application each. The most common topics of applications related to obesity, including diet, weight and physical activity (15 in total), with the majority focused on adults or whole populations.

The findings of the document analysis informed the interview schedules for practitioners and researchers, as described in the second work package above.

### Interviews

Overall, the interview findings have been framed as a 'SWOT’ (strengths, weaknesses, opportunities and threats) analysis of the PHPES. This framework was chosen as it provided a strategic tool for organising the themes from the analysis interview data, which facilitated the development of actionable recommendations for the SPHR Executive Board for the relaunch of the scheme (see 'Discussion’ section). We will discuss each element in turn below.

*Strengths*: PHPES provides much needed resources for evaluation which often are not available locally, and produces useful evidence to understand where a programme is not delivering, which can be used to formatively develop interventions.

'Because we’re under such a lot of pressure, I think, in terms of how you allocate your resources and who’s doing what to their capacity, or do you lose posts. Having this piece of research strengthens your case to say that, look, this piece of public health involvement enlightens things, it’s well-evidenced and we should leave it alone rather than trying to mess with it and to say that it’s not a priority.’ *(Practitioner, funded project)*

Practitioners benefit from academics with specific knowledge within their field, and relationships between practice and academia are strengthened.

'It was not only towards the end of the project, but it was throughout the project that we were really into the intelligence of how the programme should be run. And not the programme that we are evaluating, but what to learn from [similar programmes] and how they should be run and what commissioners should think about when they are commissioning new programmes, if you like…. So, I think that has had a major impact into their decisions to fund the programmes in future.’ *(Academic, funded project)*

We found evidence in our evaluation that PHPES had generated some valuable case studies/exemplars of effective local evaluations with significant impact. In this study we did not set out to measure impact, and therefore we were dependent on what project teams reported (see examples provided above of the Sheffield Housing+ programme and the St Ives Falls Prevention Pilot in Cambridgeshire). However, all delivered projects identified local impact in their project reports.

*Weaknesses*: There may be a need to widen the objectives of PHPES beyond (cost-)effectiveness of interventions to address the needs of practitioners, such as evaluations focusing on the implementation of programmes. Currently, the scheme only funds evaluation of the effectiveness of interventions, while practitioners expressed a need for process evaluations of interventions that are currently being implemented and support in adapting and implementing evidence-based interventions in their local context.

'When the original bid went in, SPHR really wanted us to do a randomised trial and methodology, and we argued quite vociferously that that wasn’t appropriate. But I think we were always trying to, trying to keep an eye on heeding what SPHR might be looking for from a methodology point of view and what was needed practically in the project.’ *(Practitioner, funded project)*

More flexibility, for example around start and end dates to take into consideration any delays in the roll-out of the intervention, setting up of the project or any contractual issues within the practitioner institution, was also requested by practitioners and academics to account for this widening scope.

'The thing I still think should, and hopefully will, happen in next round is, in the end, it wasn’t an evaluation. Sorry, there wasn’t an intervention to evaluate. Because there was nobody recruited. And there are other projects that I know that just haven’t done what they are supposed to, because of delays, for example. Which is, in a way, a similar problem…. So, I think the main, big learning from this particular project is that rather than submitting a full evaluation thing and having set times on which you have to start, on which you have to do things, either it has to be more flexible, depending on what happens in practice.’ *(Academic, funded project)*

The need for flexibility relates to differences in timescales between academic research and public health decision-making, with specific ‘windows of opportunity’ [16] for inputting evidence that often do not align with the rigour of the research process, as emphasised in the literature [15].

Finally, there was a request for more feedback on unfunded projects. Even if applications to the scheme were unsuccessful, practitioners and academics valued feedback on their proposals to improve future submissions and utilise the academic expertise from reviewers to improve their monitoring and self-evaluation of interventions.

'If I know there is more transparent feedback on learning and a proper guideline that comes with the proposal, which I think is quite important, and maybe a scoring rate so when we apply, we know exactly what we need to meet and where we fail to meet that, because that’s what the MRC (Medical Research  Council)  does, they score and they explain to you where you’re scoring lower and higher. And it will be time-consuming for the funders, but I hope they understand it’s important for people who spend months. Especially if you go to the second phase, maybe it’s going to be too much for them if they have many applications in the first round, but after the second round it’s important to get more precise feedback, I think.’ *(Academic, unfunded project)*

We found no specific information in guidance documents provided by SPHR to PHPES on feedback, which might be illustrative of a lack of attention to feedback mechanisms in the development of the scheme. However, previous studies have highlighted that these mechanisms are a foundation stone for collaborative research and can help to maintain relationships between PHPs and academic research. Where these connections were lacking, this resulted in a focus on knowledge production and transfer, rather than co-production [17].

*Opportunities*: Better-developed interventions were more likely to be funded. Therefore, the scheme could encourage novel/promising but less developed ideas. To support these ideas, funded time to develop a protocol and ensure feasibility of the intervention prior to application could increase intervention delivery success rates.

'I think the short form was great to get things going, and identify promising ideas, but actually then you need 6 months to write a proper protocol. And if we’d had that time, you’d realise that all the things that this agency, this intervention, were promising, actually weren’t there.’ *(Academic, funded project)*

Participants were in favour of a two-stage application process to select the most promising and novel ideas.

'It is the old-style R&D [research and development], you know? We are reinventing how NIHR started. But we’ve got to remember why it stopped, which was that a lot of money was wasted on things that weren’t any good. So we’ve got to have a bit of a more critical evaluation and have a two-stage process whereby we can sort of knock on the head things which aren’t going to be any good.’ *(Academic, funded project)*

Participants also identified potential for working with regional public health research hubs in collaboration with Public Health England and the National Institute for Health Research, either face-to-face, for instance through workshops, or online through phinder, a digital portal that connects public health practice and research, to advertise ideas for research collaborations.

*Threats*: In the interviews, some participants expressed concerns that there may be a risk of increasing inequalities, as applications clustered around SPHR partner institutions.

'So I think there’s a danger—dare I even mention equality?—as to those boroughs that have invested in their public health team…they will benefit from PHPES [...] And you will know that in some public health departments they have one consultant. It’s like, why, when other public health departments have maybe four consultants… So, that sort of variation means that for the smaller departments it is quite difficult, if they’re fire-fighting, to have the time and the energy and the headspace to even think about academic work. And if you can’t think about academic work, then the whole bit about… my earlier bit about improving practice and improving what we do locally, you don’t get as much of an opportunity.’ *(Practitioner, funded project)*

Some researchers feel there may be pressure of practitioners’ organisations to show 'success’ of the intervention. There can be tensions between researchers and practitioners, for example on the use of existing research evidence and the importance of generalisability of findings and of generating peer-reviewed publications.

'It’s a bit about a culture clash between the world of academia and public health practice in many ways. So some of it is a bit clichéd in about timescales and academic purity, value, pragmatism and all that, so there’s a bit of battle in there… And so colleagues at [this LA] who are living and breathing delivery of the programme day to day, I think really struggled working with the academics. Because whilst they were absolutely up for the notion of having an independent robust academic evaluation of the programme done, they were also deeply anxious about what it would say to ensure that, obviously wanting it to clearly show the positive impact of the programme, I guess, to be honest.’ *(Practitioner, funded project)*

In sum, our SWOT analysis indicates that the PHPES scheme provides much needed resources for evaluation, with practitioners benefitting from academics with specific knowledge within their field. However, participants criticised the limited scope of the scheme (cost-effectiveness of well-established intervention) and identified opportunities for early engagement and support for PHP and academics, with a wider and more flexible focus that would also help to address threats of unequal access to support from SPHR centres. These interview findings were used to generate specific questions for the online survey and were shared at the national workshop held on 19 September.

### Online survey

The online survey was completed by 19 people before the closing date of 1st October. Eight respondents (42%) had been previously involved in PHPES. Both practitioners (4) and researchers (9) engaged in the first and second phases of SPHR completed the survey. Researchers were linked to various member organisations, including the Universities of Bristol, Exeter and Sheffield, University College London (UCL), the LiLaC collaboration between the Universities of Liverpool and Lancaster, and the Fuse [Centre for Translational Research in Public Health] collaboration.

Respondents thought that meetings between practice partners and academics prior to developing PHPES applications would most likely increase the chances of successful research collaborations. These meetings could help to clarify expectations about the contributions of each partner in the project and what to do when these expectations were not met (conflict resolution). Giving practitioners an active role in the research process would also make collaborations more likely and successful. These factors were mirrored in the responses to the question of what makes collaborative research more unlikely, with no engagement between practitioners and researchers prior to submission, lack of support from academics and no active involvement from practitioners in the research process deemed as reducing the likelihood of successful collaborations. In addition, the evaluation of interventions that were still under development or implemented only recently were judged to impact negatively on the success of PHPES projects (see Table 2).

**Table 2:** What makes successful research collaborations between PHPs and academics likely and unlikely? (*n* = 19)

Successful research collaboration likely if	*N*	Percent of cases	*N*	Percent of cases	Successful research collaboration unlikely if
Clear guidance is available on the process and timescales for applying for PHPES funding	5	31.3	7	43.8	Guidance on the process and timescales for applying to PHPES are unclear
Support is available from academic researchers within SPHR for developing the research idea and checking whether the idea is eligible and feasible for evaluation	6	37.5	8	50.0	No support is available from academic researchers within SPHR for developing the research idea and checking whether the idea is eligible and feasible for evaluation
The intervention suggested for evaluation has been clearly defined and has been implemented for at least a year, and providers are supportive of the evaluation	5	31.3	8	50.0	The intervention suggested for evaluation is still under development, it has not been clearly defined yet, has not been implementation so far, and lacks support from potential providers for the evaluation
Practice partners and academic researchers meet up to discuss the idea for evaluation before they develop the application	12	75.0	13	81.3	Practice partners and academic researchers do not meet up to discuss the idea for evaluation before they develop the application
Practitioners and academic researchers already have established relationships from previous collaborations	6	37.5	2	12.5	Practitioners and academic researchers involved in the application have not worked together before
Expectations about what each partner will contribute and what they will get out of the project have been clarified, including how potential conflicts will be resolved	8	50.0	7	43.8	Expectations about what each partner will contribute and what they will get out of the project are not made clear in the application
Practitioners have an active role in the research project, for example, co-design the research questions and collect data as peer researchers	7	43.8	8	50.0	Practitioners are not involved in the delivery of the research. For example, the research questions are decided by the researchers, and the data are only collected by the research team
Academic researchers are co-located in the practice organisation for the duration	2	12.5	2	12.5	Academic researchers are not co-located in the practice organisation for which the evaluation is being conducted
Outputs and dissemination activities are identified from the start, with clear involvement from wider stakeholders/knowledge users	6	37.5	3	18.8	Outputs and dissemination activities are not identified from the start and do not involve wider stakeholders/knowledge users in these activities
Clear and timely feedback is provided on proposals to all applicants. including signposting to other funding opportunities	1	6.3	1	6.3	Feedback is not provided on proposals to all applicants, and unsuccessful applicants are not signposting to other funding opportunities
The collaboration leaves a legacy that sustains the intervention	6	37.5	4	25.0	The collaboration does not aim to leave a legacy for sustaining the intervention
Funding can be used partly towards intervention costs	5	31.3	4	25.0	Funding is only available for research (not for intervention costs)
Funding can be used for evidence reviews, secondary data analysis and network development	3	18.8	4	25.0	Funding can only be used for evaluation of local interventions
Start and end dates of project are flexible to account for delays in start of interventions and any contractual issues	5	31.3	4	25.0	Start and end dates of projects are fixed and cannot be changed to account for delays in start of interventions and any contractual issues
Total	77	481.3	75	468.8	

**Fig. 1:**
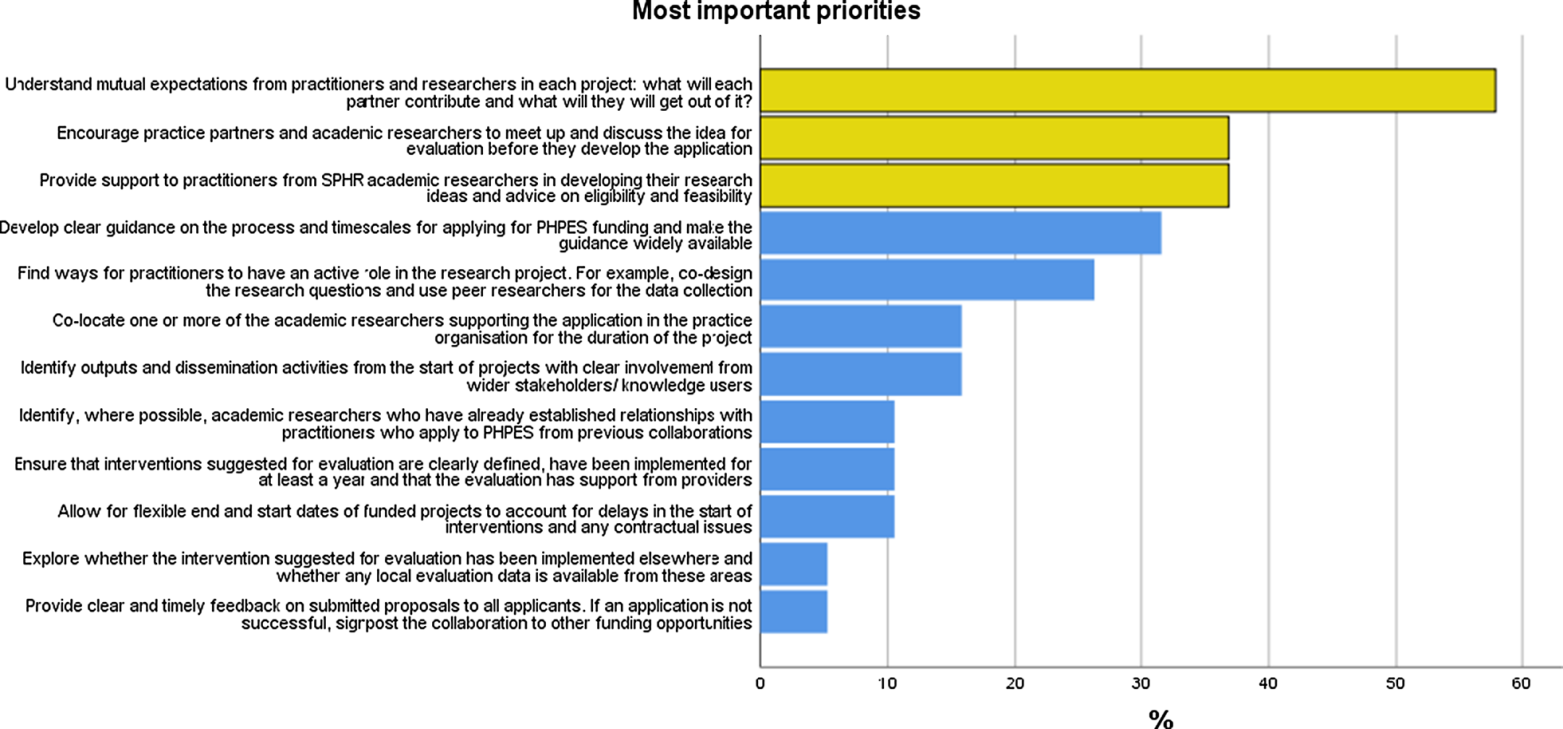
Priorities for collaborative research between PHPs and academics (*n* = 19)

Therefore, the top three priorities emerging from the survey centred on facilitating mutual understanding of expectations by encouraging practitioners and academics to meet up before submitting proposals, and providing practitioners with support from member organisations to work up their ideas into feasible projects on sufficiently established public health practices (Fig. 1). Most respondents agreed that dedicated support from an SPHR member was essential to developing ideas into feasible submissions. The strongest disagreement focused on whether only practitioners should be allowed to submit ideas to PHPES, with almost all respondents agreeing that researchers should also be able to present their suggestions for research.

### National workshop

The results of the online survey were verified and expanded upon in the national workshop. The workshop discussions focused on three questions around the key objectives, scope and scale of PHPES.

*Key objective*: Participants recommended a balance in funded projects that generated both local and generalisable knowledge. Transferability of knowledge was suggested as a key selection criterion for future projects.

*Scope:* Expand the focus beyond effectiveness of intervention studies to include implementation research and qualitative process evaluations, but linked to the new SPHR themes to identify available expertise within member organisations.

*Scale*: Fund a mix of large-scale (comparative) projects and small-scale projects in favour of a larger number of small-scale projects to ensure good spread of projects across regions and member organisations.

## Discussion

In this paper we reviewed the learning from PHPES (2013–2017) and use this to develop recommendations for a revised scheme within the current SPHR programme (2017–2022). We frame our discussion of the study findings in the wider context of similar funding schemes for which the lessons learnt from this evaluation are also relevant.

### Key findings: factors making applications more successful

The combined findings from the document analysis, interviews and online survey and workshop suggest that the PHPES is valued by both practitioners and researchers. However, participants criticised the limited scope of the scheme (cost-effectiveness of well-established intervention) and identified opportunities for early engagement and support for PHP from academics, with a wider and more flexible focus that would also help to address threats of unequal access to support from SPHR centres.

Our evaluation of PHPES highlights that the success of collaborative research projects between public health practitioners (PHP) and researchers can be improved by organising regional development workshops to explore the feasibility of ideas and clarify expectations; providing variable levels of funding to projects at different stages of development, including smaller scoping studies; and by dedicating more resources for disseminating findings across the PHP community nationally, as well as to practice partners involved in the projects locally.

### Three tensions in collaborative research projects

Our study also indicates that collaborative research projects can increase tensions between practitioners and researchers. We discuss three tensions below, related to (1) the scope of collaborations, (2) local versus national impact and (3) increasing inequalities in access to funding.

#### Scope of collaborations

Applications were more successful if they evaluated the (cost-)effectiveness of more developed interventions, while evaluation of more novel but less well-established interventions were less likely to be funded. While this is understandable from a risk management perspective (interventions under development might also be harder to evaluate), practitioners emphasised a need for risk-taking and experimenting with new types of interventions despite the lack of evidence to support them. PHPES can provide a unique opportunity for funding the evaluation of these projects, to develop their evidence base (including proving that they are not worthy of further development), before applying to other funding programmes for scaling up the interventions, which often demand an established evidence base and proof of concept.

This tension also threw up questions about the scope of PHPES: cost-effectiveness analysis was more often successful but not always feasible for the novel interventions and evidence needs that practitioners expressed through our research. Practitioners welcomed a widening of the scope of PHPES to include qualitative process evaluations and implementation studies to support the development of less established interventions.

Although the eligibility criteria in guidance documents provided to applicants do not explicitly state that other types of projects are not allowed,^1^ the guidance assumes that applicants will be most interested in cost-effectiveness evaluations of existing interventions by stating this explicitly in the PHPES offer ‘to provide evidence on the cost-effectiveness of your work that others can use’, which has potentially steered applicants in this direction.

The limited scope of PHPES points to differences between PHPs and academic researchers in the type of evidence that is valued. This finding builds on previous studies stressing the need for constant alignment of values and expectations around evidence between PHPs and academics [18], with our study suggesting a widening of scope for evidence collected and research methods in research collaborations.

#### Local versus national impact

Related to this question was another tension about maximising the impact from collaborative projects. Practitioners were keen for the project to provide them with evidence for local impact: how to make the project work here, how can we change local practice and inform local policies? Researchers, on the other hand, wanted to ensure that findings of projects were generalisable at the national level and focused on publishing the research in national and international academic journals, which were deemed much less relevant for dissemination by practitioners. This led to tensions between practitioners and researchers on some projects about the need for generalisable findings, where to publish them and how much use existing research evidence would be for the project.

These tensions relate to differences in expectations about what counts as evidence and how research findings will be used, illustrating the need for expectation management from the start of collaborative research and taking time to build a ‘collaborative identity’, as suggested in previous studies [18].

The participants at the national workshop who reflected on these issues suggested that to maximise the potential impact of PHPES projects, applications should explicitly outline both the relevance to the local context and the potential generalisability of the findings at a national level. This concept of transferability was suggested as a means of capturing both dimensions: relevance to local context and potential for application elsewhere. This could be included as a criterion for funding in future PHPES rounds. In addition, more resources within the scheme could be dedicated to dissemination and mobilisation of findings across the public health practice community nationally, as well as practice partners involved in the projects locally.

#### Increasing inequalities in access to funding

Finally, successful applications appeared to cluster around SPHR partner institutions, based on geographical proximity of applicants to researchers, which made it easier for practitioners to approach researchers with ideas for applications and gain advice on the feasibility and potential design of suggested evaluations. However, this has the potential to increase inequalities in the geographical distribution of research funding and missed opportunities to evaluate novel interventions developed in locations further from academic public health departments. Therefore, workshop participants suggested provision of regional evaluation development workshops for those interested in applying to PHPES or other sources of funding to explore the feasibility of ideas and clarify expectations between practitioners and researchers. Regional development workshops could be held to facilitate prior development of ideas between researchers and practitioners before formally applying to PHPES and could be supported by regional public health research hubs (developed and supported by regional PHE centres) and advertised via the NIHR phinder website.

These workshops could also help to build relationships and trust between practitioners and researchers, which are key to the success of any collaboration [17]. Developing these relationships before a project is funded may be equally important as ensuring a good working relationship during the project. Opportunity for early discussions may determine whether tensions can be resolved or are allowed to threaten effective delivery and dissemination of a project.

### How do other funders address these tensions?

In addressing questions about scope, impact and equality, PHPES could learn from other funding schemes across the United Kingdom that aim to facilitate collaborative research between practitioners and academics working in public health.

#### NIHR PHIRST

NIHR recently launched a call for local authorities to submit initiatives for evaluation by two nationally operating responsive research teams [22]. The Public Health Research Programme appointed two academic teams (the ‘PHIRST teams’) that are ready to work with local authorities on priority initiatives. The academic teams are fully funded to co-design and undertake robust and independent research in partnership with local authorities and their partners (NIHR PHR).

The scheme addresses the tension of inequality of applications by requiring the teams to operate on a national scale, and not only on a geographical basis with partners that they are already working with. The onus is put on the responsive academic teams to demonstrate 'the team’s geographical reach and understanding of structures within countries and across countries’ [22]. Team are required to develop a strategy for how to respond if they are asked to conduct a piece of research in a geographical location they are less familiar with. The ability to work across the United Kingdom and not just within the team’s immediate geographic location is an essential requirement of the scheme and supports less well connected local authorities in developing collaborative research proposals with academic researchers.

Although new projects are funded by PHIRST on an annual basis, evaluations can take place over the 3-year lifespan of the teams, providing flexibility to evaluation start and end dates to both local authorities and the researchers, depending on changes in local contexts.

#### HRB APA

Another example is the Applied Partnership Awards (APA) scheme that is operated by the Health Research Board in Ireland (HRB) [23]. APA awards encourage partnership-based, co-funded research applications, led by a knowledge user from a practice organisation to address a nationally relevant issue that can be applied to the knowledge user organisation within two years. The scheme requires that knowledge users are involved as active partners throughout the research process and that the knowledge users are willing to invest time and resources to the successful completion of the research. A unique feature of this award scheme is that salary-related funding may be requested from the HRB to enable the release time for knowledge users.

The scheme solves the tension of scope identified in this study by requiring applicants to develop research projects in response to nationally relevant priority areas; however, the teams of researchers and knowledge users need to ensure that the findings from the research have a direct impact on the decision-making of the lead knowledge user’s organisation. Therefore, the team has to provide documented evidence in their application, demonstrating that the proposed research is explicitly linked to evidence needs of the knowledge user organisation.

Moreover, applications need to include a clear and concise knowledge translation plan that highlights how the research findings will be applied by the knowledge user organisation. This solution also addresses the tension of local versus national impact by prioritising local impact that is explicitly linked to national priorities and hence has relevance beyond the local context.

Both schemes hold important lessons for funders on how to address existing and ongoing tensions in developing research by asking research teams to develop clear strategies for responsive research beyond their geographical comfort zones, and by balancing a focus on national priorities with findings that can be directly implemented in local organisations through a dedicated knowledge translation plan and buy-out of time for lead knowledge users.

### What happened next with SPHR PHPES?

All members have been active participants in PHPES applications in the past before joining the evaluation team for the PHPES project. During the evaluation, we feed back emerging findings to the SPHR Executive Board, which include both academic and practice partners, to inform their thinking about the design and launch of PHPES in future rounds.

The recommendations made in the final report of the PHPES evaluation submitted to the SPHR Executive Board have been implemented in the redesign of the PHPES call launched in February 2019. For example, an additional stage to support regional brokerage between practitioners and SPHR members in order to explore the feasibility of ideas and mutual expectations between partners was introduced in the application process. In addition, the scope of the scheme was broadened, as recommended in our report, to include a wider range of research designs, such as implementation projects and process evaluations.

We helped to significantly revise the guidance for PHPES applicants following the evaluation recommendations and promoted the redesigned scheme and guidelines at the SPHR Annual Scientific Meeting through a poster display and interactive workshop for both researchers and practitioners. During the workshop, five practice partners pitched their ideas for PHPES application, which were subsequently taken forward in follow-up discussion with SPHR members.

To support the additional regional brokerage stage, we organised two regional PHPES workshops (one in Sheffield and one in Newcastle) to support early conversations between practitioners and researchers about ideas for applications, which were well attended and resulted in various applications being submitted to stage 1 of the scheme. Finally, research team members joined as members of the national panel, which reviewed 16 submitted stage 2 applications, supporting the panel with insights from the research.

While the evaluation generated valuable recommendations for improving the design and delivery of PHPES, active involvement from the research team in disseminating and implementing the findings (such as drafting new application forms and guidance, organising regional development workshops, reviewing new applications) proved essential to the effective implementation of the recommendations. This holds true for the PHPES itself: active engagement between researcher and PHPs before, during and after the development and delivery of a study is important in maximising the potential for having an impact on local PH practice.

### Strengths and limitations

We found it challenging to involve practitioners in the research at all levels, particularly those who had been involved in applications that had not been funded. Even for funded projects, a number of the practice partners had left the position they held at the time of the PHPES project and could not be contacted. Other practitioners, despite planning to attend the national workshop, had to cancel due to other commitments on the day. They were replaced by lay participants who were recruited from a local public involvement panel established to support public health research. This ensured lay input into the development of the project as well as input into the scope of the findings.

The focus of the PHPES evaluation and of this paper is on the experiences of PHPs and academic researchers engaging in collaborative research through the scheme, and therefore limited data were included on the perspective of the funder (such as SPHR core staff). One of the interviewed academics was also the lead for PHPES within SPHR; however, the interview focused on her experiences in projects within the scheme and not on the operation of the scheme itself. As a result, data were not available on the rationale for the scope and feedback mechanisms in PHPES, which were identified as tensions (weaknesses) in this study. We have tried to address this gap by reviewing guidance documentation provided to PHPES applications over the years. However, we found limited data on the funder perspective in these documents to provide insight on the identified tensions. Future research could more clearly include the funders’ perspectives when evaluating similar schemes.

## Conclusions

Our study highlights that the PHPES, a responsive funding scheme, provides much needed resources for evaluation which often are not available locally, and produces useful evidence which can be used to formatively develop interventions. Practitioners benefit from academics with specific knowledge within their field, and relationships between practice and academia are strengthened.

Furthermore, our evaluation suggests that the success of collaborative research applications between PHP and researchers can be improved by the following: organising regional development workshops to explore the feasibility of ideas and clarify expectations (which reduce inequality in success rates); providing variable levels of funding to projects at different stages of development, including smaller scoping studies (to increase the scope of collaborations with a focus on transferability of findings from local to national contexts); and dedicating more resources for disseminating findings across the PHP community nationally, as well as to practice partners involved in the projects locally. This makes it possible to support both transferability and ongoing relationship-building between academics and practitioners.

The implementation of these recommendations in the relaunch of PHPES in February 2019 suggests that the scheme has been successful in increasing access to the scheme, widening the scope of collaborations and improving the potential transferability of research findings, with a better balance between local priorities and national relevance, based on earlier conversations between practitioners and academics.

Funders that are keen to support collaborative research between PHP and academics need to be mindful of three tensions in developing applications, related to (1) the scope of collaborations, (2) local versus national impact and (3) increasing inequality in access to research funding. Our study and comparisons with related funding schemes demonstrate how these tensions can be successfully addressed by providing practical solutions. These solutions illustrate how differences in expectations, values, processes and structures can be aligned between the PHP and academic. The need for this alignment has been identified in previous studies but limited evidence is available on how to do this in practice, particularly in the context of funding research collaborations at a local level.

## Supplementary Information

**Additional file 1: File 1.** Data extraction template for document analysis. **File 2.** Interview schedule for SPHR practitioners. **File 3.** Interview schedule for SPHR researchers. **File 4.** Online survey. **File 5.** Programme national workshop.

## Abbreviations

HRB APA: Health Research Board Applied Partnership Awards
LA: Local Authorities
MRC: Medical Research Council
NIHR PHIRST: National Institute for Health Research Public Health Intervention Responsive Studies Teams
NIHR SPHR: National Institute for Health Research School for Public Health Research
PHP: Public health practitioners
PHPES: Public Health Practice Evaluation Scheme

## Footnotes

1 To be eligible for SPHR PHPES funding, applications need to aim to improve population health and/or reduce health inequalities, have the potential to be of benefit in other parts of England, and have secure funding for the duration of the evaluation (normally around 2 years),

## Availability of data and materials

The data sets, which include anonymised interview transcripts and a description of our coding trees, are available from the corresponding author on reasonable request.

## References

[1] Innvær S, Vist G, Trommald M, Oxman A (2002). Health policy-makers' perceptions of their use of evidence: a systematic review. J Health Serv Res Policy.

[2] Lomas J (2000). Essay: using ‘linkage and exchange’ to move research into policy at a Canadian foundation: encouraging partnerships between researchers and policymakers is the goal of a promising new Canadian initiative. Health Aff.

[3] Greenhalgh T (2019). How to read a paper: the basics of evidence-based medicine and healthcare.

[4] Ogilvie D, Craig P, Griffin S, Macintyre S, Wareham NJ (2009). A translational framework for public health research. BMC Public Health.

[5] Graham ID, Tetroe JM (2009). Getting evidence into policy and practice: perspective of a health research funder. J Can Acad Child Adolesc Psychiatry.

[6] Kitson AL, Rycroft-Malone J, Harvey G, McCormack B, Seers K, Titchen A (2008). Evaluating the successful implementation of evidence into practice using the PARiHS framework: theoretical and practical challenges. Implement Sci.

[7] French B, Thomas LH, Baker P, Burton CR, Pennington L, Roddam H (2009). What can management theories offer evidence-based practice? A comparative analysis of measurement tools for organisational context. Implement Sci.

[8] Moore G, Todd A, Redman S. Strategies to increase the use of evidence from research in population health policy and programs: Evidence Check rapid reviews brokered by the Sax Institute (http://www.saxinstitute.org.au) for the Primary Health and Community Partnerships Unit, NSW Department of Health; 2009.

[9] Oliver K, Everett M, Verma A, de Vocht F (2012). The human factor: re-organisations in public health policy. Health Policy.

[10] Contandriopoulos D, Lemire M, Denis JL, Tremblay É (2010). Knowledge exchange processes in organizations and policy arenas: a narrative systematic review of the literature. Milbank Q.

[11] Van Der Graaf P, Forrest LF, Adams J, Shucksmith J, White M (2017). How do public health professionals view and engage with research? A qualitative interview study and stakeholder workshop engaging public health professionals and researchers. BMC Public Health.

[12] Holmes B, Best A, Davies H, Hunter D, Kelly M, Marshall M (2017). Knowledge-to-action in complex health systems: who should do what?. Evid Policy.

[13] Al Hallami M, Brown C (2014). Scenarios of London local authorities' engagement with evidence bases for education policies. Issues Educ Res.

[14] Kneale D, Rojas-García A, Raine R, Thomas J (2017). The use of evidence in English local public health decision-making: a systematic scoping review. Implement Sci.

[15] Atkins L, Kelly MP, Littleford C, Leng G, Michie S (2017). Reversing the pipeline? Implementing public health evidence-based guidance in English local government. Implement Sci.

[16] Brownson RC, Chriqui JF, Stamatakis KA (2009). Understanding evidence-based public health policy. Am J Public Health.

[17] Rycroft-Malone J, Burton CR, Wilkinson J, Harvey G, McCormack B, Baker R (2015). Collective action for implementation: a realist evaluation of organisational collaboration in healthcare. Implement Sci.

[18] Kislov R, Harvey G, Walshe K (2011). Collaborations for Leadership in Applied Health Research and Care: lessons from the theory of communities of practice. Implement Sci.

[19] NIHR SPHR. Public Health Practice Evaluation Scheme (PHPES). 2020. https://sphr.nihr.ac.uk/get-involved/public-health-practice-evaluation-scheme-phpes/?cookiebanner=true.

[20] Srivastava A, Thomson SB. Framework analysis: a qualitative methodology for applied policy research. 4 J Admin Govern. 2009;72.

[21] SPHR N. Learning from the first five years of NIHR School for Public Health Research: a public involvement perspective. 2020. https://sphr.nihr.ac.uk/research/capturing-the-learning-from-public-involvement-in-the-first-five-years-of-the-nihr-school-for-public-health-research/.

[22] NIHR PHR. Public Health Intervention Responsive Studies Teams (PHIRST) call for Local Authority Initiatives. 2020. https://www.nihr.ac.uk/funding/public-health-intervention-responsive-studies-teams-phirst-call-for-local-authority-initiatives/24248.

[23] HRB. Applied Partnership Awards (APA) 2019. 2020. https://www.hrb.ie/funding/funding-schemes/all-funding-schemes/grant/applied-partnership-awards-apa-2019-next-call-tbc/.

