# Supplementary material for: What makes a ‘successful’ collaborative research project between public health practitioners and academics? A mixed-methods review of funding applications submitted to a local intervention evaluation scheme
Source: Health Res Policy Syst. 2021 Jan 20;19:9. doi: 10.1186/s12961-020-00671-0 (PMC7816377; doi:10.1186/s12961-020-00671-0)
Supplement: Supplementary file 1 — Additional file 1: File 1. Data extraction template for document analysis. File 2. Interview schedule for SPHR practitioners. File 3. Interview schedule for SPHR researchers. File 4. Online survey. File 5. Programme national workshop. [file 12961_2020_671_MOESM1_ESM.docx]

**Additional files**

File 1. Data extraction template for document analysis

File 2. Interview schedule for SPHR practitioners

File 3. Interview schedule for SPHR researchers

File 4. Online survey

File 5. Programme national workshop

1. **Data extraction template for document analysis**

| **Reference** |  |
| --- | --- |
| **Title** |  |
| **Funded y/n** |  |
| **Funding required** |  |
| **Round?** |  |
| **SPHR partner named?** |  |
| **Date/timeframe** |  |
| **Location** |  |
| **Topic SPHR theme** |  |
| **Project outline and objectives** |  |
| **Health and other outcomes** |  |
| **Target population and reach** |  |
| **Effectiveness evidence – impact on health inequalities** |  |
| **Advice/initial contact from SPHR** |  |
| **Partner contribution** |  |
| **Other information** |  |

FUNDED APPLICATIONS ONLY

| **Ref /short title** |  |
| --- | --- |
| **Completed on time Y/N** |  |
| **Outputs**  **Dissemination** |  |
| **Info on outcomes, impact,**  **Additional grants?** |  |
| **Relationship with partner (amount of contact etc.)** |  |
| **Objectives/ milestones**  **Achieved?** |  |
| **PPI involvement** |  |
| **Other relevant info** |  |

1.
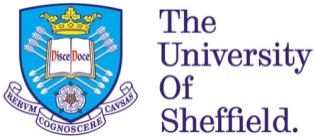
**Interview schedule for SPHR practitioners**


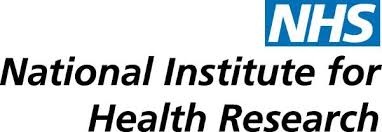
**TOPIC GUIDE
For interviews with SPHR practitioners**

**Introduction**Thank-you again for agreeing to take part in this research project.

We are interested in finding out your experiences of applying to the School of Public Health Research’s (SPHR), Public Health Practice Evaluation Scheme (PHPES). I will be asking you a few questions about the scheme, and we are really interested in your views.

- Everything you say will be confidential. No names will appear on any reports and you will not be able to be identified.
- With your permission, I will tape record the interview.
- If there are questions you do not wish to answer, then you don’t have to
- You can stop the interview at anytime

Do you have any questions? Have you provided us with a consent form? [*Sent this via email prior to interview]*

1. **Background
   To begin I would just like to ask you a few questions about you and your working history [5 min]:**

- In what position and what institution are you currently working?
- Could you tell me about your prior working history? Have you always been a public health practitioner?
- Have you got previous experience of working with academic researchers on research projects? *Ask to describe extent of this…. is this partnership with academia encouraged within their institution?*

1. **PHPEs application process**

**I would now like to ask you about the process of applying to the PHPES scheme [10 min]:**

- How did you originally hear about the PHPES scheme?
- How did you find the process of applying to the PHPES scheme? *Prompt: Were the instructions clear to you? How did you find using the form?* *How could the process be improved? Were you given enough time to develop and submit an application?*
- How much prior contact did you have with your academic contact before applying to the PHPEs scheme? *Prompt:* Did you know your academic partner prior to applying to the scheme? How did you get In touch with the academic partner? *Who instigated this contact originally? Did the original idea come from you, and if so did you initially approach the academic partner about the project (or did they approach you)?*
- Did this prior contact help you with the application process?
- How did you go about developing the proposal with your academic partner? *Prompt: did they help draft the form?*

**I am now going to ask some questions about your specific PHPES project [10 min]:**

**UNFUNDED PROJECTS**

**•** Did you receive feedback on your unfunded application? How helpful did you find this process? Prompt: did the feedback appropriately demonstrate why the research was not funded? Did the feedback help you to improve your research idea; and

• Did you apply for funding elsewhere?

**FUNDED PROEJCTS:**

- Did the project start at the intended time, were the original aims and objectives achieved (*if not, why not*)
- How have the project outputs been used within your institution? *Prompt: project report, journal articles, case studies*
- Has the project lead to any changes in policy or practice within your institution? (e*xample ?)*

1. **Relationship with academics [10 min] FUNDED PROJECTS ONLY:**

**FUNDED PROJECTS ONLY: I am now going to ask you some questions about the relationship between the academic and practitioner partner in your project:**

- - - How did you find working with academics on your research project?
    - How did the day to day management of the project work in practice?
    - How often did you meet/have contact with your academic partner?
    - Did you feel you were able to build up a relationship?
    - Were there any barriers/difficulties?
    - What were the highlights?

1. **Conclusion**

**I am going to finish by asking you some concluding questions now [5 min]**

- Would you apply for the PHPES scheme again? Why/why not? Would you do anything differently?
- Now the project has finished/now the application process is over (delete as appropriate), are you still in contact with your academic partner?
- Would you work with your academic partner again?
- Do you think that PH professionals and academics mean the same thing when they talk about evidence?
- What can SPHR do to improve the PHPES scheme. What type of academic support are you most interested in?

**We’ve covered a great deal, before you finish, is there anything else that you think is relevant? Do you have any questions to ask me? Would you like to be informed about the findings of the research? We will contact you by email at the end of the project.**

1. **
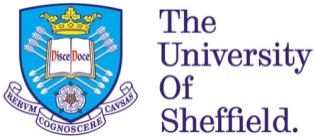
Interview schedule for SPHR researchers**


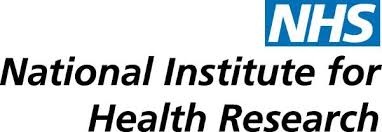
**TOPIC GUIDE
For interviews with SPHR researchers (academics)**

**Introduction**Thank-you again for agreeing to take part in this research project.

We are interested in finding out your experiences of applying to the School of Public Health Research’s (SPHR), Public Health Practice Evaluation Scheme (PHPES). I will be asking you a few questions about the scheme, and we are really interested in your views.

- Everything you say will be confidential. No names will appear on any reports and you will not be able to be identified.
- With your permission, I will tape record the interview.
- If there are questions you do not wish to answer, then you don’t have to
- You can stop the interview at anytime

Do you have any questions? Have you provided us with a consent form? *Send this via email prior to interview?*

1. **Background**

**To begin I would just like to ask you a few questions about you and your working history [5 min]**

- In what position and what institution are you currently working?
- Have you got previous experience of working with practitioners on research projects? *Ask to describe extent/ example of this…. is this partnership encouraged within their institution?*

1. **PHPEs application process**

**I would now like to ask you some general questions about the process of applying to the PHPES scheme [10 min]**

- How did you originally hear about the PHPES scheme?
- How did you find the process of applying to the PHPES scheme? *Prompt: Were the instructions clear to you? How did you find using the forms?* *How could the process be improved?* *Were you given enough time to develop and submit an application?*
- How much prior contact did you have with your practice partner before their application to the PHPEs scheme? *Prompt:* Did you know your practice partner prior to applying to the scheme? *Who instigated this contact originally? Did the original idea come from you, and if so did you initially approach the practice partner about the project (or did they approach you)?*
- How did you support the practice partner in developing and submitting their application?

**I am now going to ask some questions about your specific PHPES project [10 min]**

**UNFUNDED PROJECTS**

- Did you receive feedback on your unfunded application? How helpful did you find this process? *Prompt:* *did the feedback appropriately demonstrate why the research was not funded?*
- Did you apply for funding elsewhere?

**FUNDED PROJECTS**

- Did the project start at the intended time, were the original aims and objectives achieved (if not, why not)
- How have you used the project outputs in your own institution? *Prompt, journal articles, news items, follow-up meetings/ collaborations, funding applications.*
- Do you feel you were able to have an impact within your partner institution?

1. **Relationship with practitioners [10 min] FUNDED PROJECTS ONLY**

**FUNDED PROEJCTS ONLY: I am now going to ask you some questions about the relationship between the academic and practitioner partner in your project:**

- - - How did you find working with practitioners on your research project?
    - How did the day to day management of the project work in practice?
    - How often did you meet/have contact with your practitioner partner?
    - Did you feel you were able to build up a relationship?
    - Were there any barriers/difficulties?
    - What were the highlights?

1. **Conclusion**

**I am now going to finish by asking you some concluding questions now [5 min]**

- Would you apply for the PHPES scheme again? Why/why not? Would you do anything differently?
- Now the project has finished/now the application process is over (delete as appropriate), are you still in contact with your practitioner partner?
- Would you work with your practice partner again?
- Do you think that PH professionals and academics mean the same thing when they talk about evidence?
- What can SPHR do to improve the PHPES scheme. What type of academic support are you most interested in?

**We’ve covered a great deal, before you finish, is there anything else that you think is relevant? Do you have any questions to ask me?** **Would you like to be informed about the findings of the research? We will contact you by email at the end of the project.**

1. **Online survey**

**Bristol Online Survey: NIHR SPHR PHPES evaluation (pre-publication version)**

*Page 1: Introduction*

***Why are we doing this survey?***

This survey is part of an internal review of the Public Health Practice Evaluation Scheme (PHPES) run by the NIHR School for Public Health Research (SPHR) between 2012-2017. The survey was developed using data from previous PHPES applications and interviews with people involved in PHPES. The aim of the survey is to gather a range of views on how we can improve the school’s PHPES. We also hope to contribute to the wider evidence base on collaborative research between public health practitioners and academics.

***Why have I been invited to complete the questionnaire?***

You have been invited to complete this survey because you are/ have been involved in previous PHPES applications or have an interest in future applications, either as a researcher, practitioner, reviewer or member of the public.

**Do I have to take part in this survey?**

You are not obliged to take part in this survey. If you do, your answers will be made anonymous and you also have the right to withdraw your response before we analyse the questionnaires (by 2^nd^ October). We will ask you to supply a made-up name so that we can find your questionnaire if you do decide to withdraw it. By filling in this form you will be giving your consent to take part in this survey. If you have any questions about the survey or the project, or would like to receive a paper copy please contact Peter van der Graaf by email: [p.van.der.graaf@tees.ac.uk](mailto:p.van.der.graaf@tees.ac.uk), or phone: 01642 342989.

The survey should take no longer than ten minutes to fill in. The closing date for the survey is midnight on **1^st^ October 2018**.

*Page 2: Question 1*

**Successful collaborative research between public health practitioners and academics in SPHR PHPES is MORE LIKELY to happen if… (Please click on up to five factors below). Please select no more than 5 answer(s).**

- Clear guidance is available on the process and timescales for applying for PHPES funding
- Clear and timely feedback is provided on proposals to both practitioners and researchers involved in the application, even if the application is not successful, with signposting to other funding opportunities
- Support is available from academic researchers within SPHR for developing the research idea and checking whether the idea is eligible and feasible for evaluation
- The intervention suggested for evaluation has been clearly defined, has been implemented for at least a year and providers are supportive of the evaluation
- The intervention suggested for evaluation has been implemented in different geographical areas or sectors with monitoring or local evaluation data available from these areas or sectors
- Practice partners and academic researchers meet up to discuss the idea for evaluation before they develop the application
- Practitioners and academic researchers already have established relationships in previous collaborations before they apply to PHPES
- Expectations about what each partner will contribute and what they will get out of the project have been clarified, including how potential conflicts will be resolved
- Practitioners have an active role in the research project, for example, co-design the research questions and collect data as peer researchers
- Outputs and dissemination activities are identified from the start with clear involvement from wider stakeholders/ knowledge users
- The collaboration leaves a legacy that sustains the intervention, either by improving the delivery model for the intervention, strengthening the business case for local funding or building local networks for continued delivery
- Funding could be used partly towards intervention costs
- Funding could be used for evidence reviews, secondary data analysis and network development

*Page 3: Question 2*

**If you would like to provide more detail about any of yours answer to question 1 or would like to add any other factors that make collaborative research between public health practitioners and academics in SPHR PHPES MORE LIKELY, please do so here.**

……

*Page 4: Question 3*

**Q3. Successful collaborative research between public health practitioners and academics in SPHR PHPES is LESS LIKELY to happen if… (Please click on up to five of the factors below). Please select no more than 5 answer(s).**

- Guidance on the process and timescales for applying to PHPES are unclear
- Feedback is not provided on proposals to both practitioners and researchers involved in the application, even if the application is successful, including signposting to other funding opportunities
- No support is available from academic researchers within SPHR for developing the research idea and checking whether the idea is eligible and feasible for evaluation
- The intervention suggested for evaluation is still under development, e.g. has not been clearly defined yet, has not been implementation so far, and lacks support from potential providers for the evaluation
- The intervention suggested for evaluation has not been implemented anywhere else or monitoring & local evaluation data is not available from similar interventions elsewhere
- Practice partners and academic researchers do not meet up to discuss the idea for evaluation before they develop the application
- Practitioners and academic researchers involved in the application have not worked together before
- Expectations about what each partner will contribute and what they will get out of the project are not made clear in the application
- Practitioners are not involved in the delivery of the research, for example, the research questions are decided by the researchers and the data is only collected by the research team
- Outputs and dissemination activities are not identified from the start and do not involve wider stakeholders/ knowledge users in these activities
- The collaboration does not aim to leave a legacy for sustaining the intervention. For example, the research only shows whether an intervention is working or not; no data is collected on financial effectiveness, wider impact and equity; and the research does not involve local networks
- Funding is only available for research (not intervention costs)
- Funding can only be used for evaluation of local interventions (not for evidence reviews, secondary data analysis and network development)
- Academic researchers are co-located in the practice organisation for the duration of the project

Start and end dates of project are flexible to account for delays in the start of interventions and any contractual issues

*Page 5: Question 4*

**If you would like to provide more detail about any of yours answer to question 3 or would like to add any other factors that make collaborative research between public health practitioners and academics in SPHR PHPES LESS LIKELY, please do so here …**

*Page 6: Question 5*

**Q5. Please tell us what are the three most important priorities for SPHR PHPES to encourage successful collaborations between public health practitioners and academic researchers in your opinion or experience. Select three priorities in the table below.**

Most important priorities

- Develop clear guidance on the process and timescales for applying for PHPES funding
- Provide clear and timely feedback on submitted proposals to both practitioners and researchers involved in the application and, if the application is not successful, signpost them to other funding opportunities
- Provide support to practitioners from SPHR academic researchers in developing their research idea and advice on eligibility and feasibility
- Ensure that intervention suggested for evaluation are clearly defined, have been implemented for at least a year and that the evaluation has support from providers
- Explore whether the intervention suggested for evaluation has been implemented elsewhere and whether any monitoring or local evaluation data is available from these areas
- Encourage practice partners and academic researchers to meet up and discuss the idea for evaluation before they develop the application
- Identify, where possible, academic researchers who have already established relationships with practitioners who apply to PHPES from previous collaborations
- Understand mutual expectations from practitioners and researchers in each project: what will each partner contribute and what will they get out of the project
- Find way for practitioners to have an active role in the research project; for example, co-design the research questions with them and involve them in data collection as peer researchers
- Identify outputs and dissemination activities from the start of projects with clear involvement from wider stakeholders/ knowledge users

*Page 7: Question 6*

**If you would like to provide more detail about any of your answers to question 5 or a different priority for successful collaborations between public health practitioners and academic researchers in PHPES please do so here.**

…

*Page 8: Question 7*

**How much do you agree with the following statements?**

|  | Strongly agree | Agree | Undecided | Disagree | Strong disagree |
| --- | --- | --- | --- | --- | --- |
| Evaluation findings that are relevant for local practice are more important that findings about whether interventions can be generalised and scaled up |  |  |  |  |  |
| PHPES projects should only address SPHR research themes (Places and Communities; Public Mental Health; Young people and children; Efficient and effective public health; Inequalities; Changing behaviour at population level) |  |  |  |  |  |
| Only interventions that have been already been implemented and have collected some form of baseline data can be eligible for evaluation |  |  |  |  |  |
| Dedicated local support from an SPHR member should be available to develop research ideas with practitioners into a feasible submission for the national panel |  |  |  |  |  |
| Researchers and practitioners should stick to what they do best and have clearly defined roles in the projects that establish boundaries between them. E.g. researchers collect and analyse the data, while practitioners provide access to participants and local intelligence |  |  |  |  |  |
| At least researchers from two SPHR member organisations should be involved in each PHPES project |  |  |  |  |  |
| Review scores of applications from the practice panel about importance for public health priorities should trump review scores from the academic panel on feasibility and generalisability of the submitted research idea |  |  |  |  |  |
| PHPES funding is best spend on many small projects across the UK to support innovative practice (instead of concentrating the funding on a small number of large research projects with the most potential for impact) |  |  |  |  |  |
| Where possible the preferred outcome of a PHPES project is a funding application for an effectiveness trial of the suggested intervention |  |  |  |  |  |
| Research ideas for PHPES can only be suggested by practitioners; academic researchers should refrain from suggesting their own ideas and contacting practitioners for their support |  |  |  |  |  |

*Page 9: Question 8*

**Do you have another other comments or suggestions about what SPHR could do to improve PHPES in the future? …**

*Page 10: Background*

**Have you been involved in PHPES applications previously?**

- Yes
- No
- Don’t know/Can’t remember

**If relevant, please tell us through which academic centre you were involved in SPHR PHPES projects (please click on that which applies)**

- Not relevant
- University of Bristol
- University of Cambridge
- University of Exeter
- Fuse (the Centre for Translational Research in Public Health)
- LiLaC (the Liverpool and Lancaster Universities Collaboration for Public Health Research)
- London School of Hygiene and Tropical Medicine
- Imperial College London
- University of Sheffield
- UCL

**Q11. Please tell us how you would describe your role (please click on all that apply)**

- Member of the public involved in the first phase of SPHR research (2012-2017)
- Member of the public already involved or hoping to be involved in the second phase of SPHR research (2017-2022)
- Researcher whose research was funded by the first phase of SPHR (2012-2017)
- Researcher whose research will be or hopes to be funded by second phase of SPHR research (2017-2022)
- Researcher who worked on a project funded by the first phase of SPHR (2012-2017)
- Researcher who will be or hopes to be working on a project funded by second phase of SPHR research (2017-2022)
- PhD student whose research was funded by the first phase of SPHR (2012-2017)
- PhD student whose research will be or hopes to be funded by second phase of SPHR research (2017-2022)
- Other

**If you selected Other, please specify: …**

*Page 11: Follow-up*

**Please give us a made-up name which will be used to identify your survey should you choose to withdraw it before the closing date of the survey.**

…

**Would you like to receive an invite to the national workshop on 19th September in Sheffield to discuss the findings and inform recommendations for the SPHR Executive Board?**

- Yes
- No

**If you answered 'Yes' to the above question, please provide us with an email address that we can send the invitation to.**

…

*Final page*

Thank you for completing this survey!

1. **National workshop programme**

**
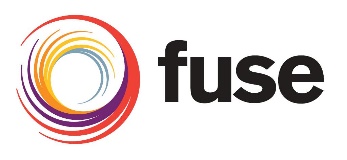

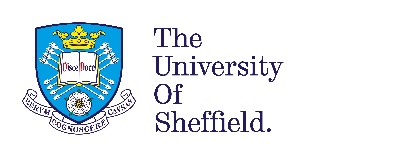
**

**NIHR SPHR Public Health Practice Evaluation Scheme (PHPES) Workshop:**

**How to improve collaborations between academic researchers and health practitioners? Developing recommendations for the future of PHPES**

**Wednesday 19th September**

**ICOSS Boardroom**

Interdisciplinary Centre of the Social Sciences

219 Portobello, Sheffield S1 4DP

https://www.sheffield.ac.uk/icoss

**10:30-2:00 (refreshments from 10am)**

**AGENDA**

1. Introductions and outline of the evaluation
2. Results of the document analysis, telephone interviews and online survey (to date)

LUNCH (provided)

1. Developing recommendations for the SPHR Executive Board in interactive table discussions
2. Next steps and dissemination activities
